# Waste-activated sludge disruption by dry ice: bench scale study and evaluation of heat phase transformations

**DOI:** 10.1007/s11356-019-05889-2

**Published:** 2019-07-09

**Authors:** Alicja Machnicka, Klaudiusz Grübel, Stanisław Wacławek, Krzysztof Sikora

**Affiliations:** 1 Faculty of Materials, Civil and Environmental Engineering, University of Bielsko-Biala, Willowa 2 STR, 43-300 Bielsko-Biala, Poland; 2 Centre for Nanomaterials, Advanced Technologies and Innovation, Technical University of Liberec, Liberec, Czech Republic; 3 Faculty of Mechanical Engineering and Computer Science, University of Bielsko-Biala, Willowa 2 STR, 43-300 Bielsko-Biala, Poland

**Keywords:** Dry ice, Waste-activated sludge, Phase transformation processes, Organic and inorganic matter, Infrared radiation

## Abstract

The freezing process consists of dissipating heat from the product until the final temperature is lower than the temperature of crystallisation of that product. Freezing can be used for numerous applications, including for disruption of waste-activated sludge (WAS). The aim of this study was to calculate the estimated amount of heat conveyed between the solidified carbon dioxide and the WAS, in the following ratios: 0.25:1; 0.5:1; 0.75:1 and 1:1. In heat of phase transformations, dry ice sublimation, water solidification, the amount of heat transferred by other substances and heat transferred from the sludge (dry sludge) were taken into account during the process of WAS freezing. Heat changes on the surface of WAS were registered using a thermovision camera. The effectiveness of WAS disintegration was confirmed by several biochemical parameters such as soluble chemical oxygen demand (increase over 14 times), degree of disintegration (48%), proteins (increase over 5 times), carbohydrates (increase almost 7 times), RNA (increase by 2.23 mg L^−1^), ammonia nitrogen (increase over 23 times), phosphates (increase almost 27 times) and turbidity (increased over 7 times). It was found that dry ice pretreatment of WAS can be an intriguing alternative for the conventional methods used.

**Electronic supplementary material:**

The online version of this article (10.1007/s11356-019-05889-2) contains supplementary material, which is available to authorized users.

## Introduction

Technological development, population growth and improvement in the quality of life bring about an increase in the amount of sewage sludge. On the other hand, the tightening of regulations forces treatment plants to search for highly efficient methods of purifying sewage and recycling sewage sludge. With the guidelines of the European Union, it is aimed to maximally reuse the sewage sludges, both on the stage of recovery of products and minimisation of sludges, as well as final management. Agricultural management of sludges is subject to strict regulations and storage requires development of a strategy, which aims to reduce the amount of biodegradable waste (Babatunde and Zhao 2007).

Over the past few years, the advantages of sewage sludge disintegration processes have been discovered, such as sewage sludge conditioning and increasing the efficiency of stabilisation processes. Disintegrative treatment methods of sewage sludges belong currently to the most dynamically developing technologies. They concern mainly the possibility of reducing the amount of sludge discharged from sewage treatment plants (Liu et al. 2016; Machnicka and Grübel 2016) and improvement of susceptibility of the sludge into a biochemical decomposition (Kardos et al. 2011; Wacławek et al. 2016; Liu et al. 2019). Disintegration is a process of destroying the structure of the sludge, which involves fragmenting of flocs, destruction of cells of microorganisms and releasing organic substances, inorganic substances and extracellular polymers into the supernatant of the sludge (Yu et al. 2008; Grübel et al. 2014). Sewage sludge pretreatment uses a variety of disintegration methods, such as high pressure (Gogate et al. 2001; Mirota et al. 2011); mechanical (Müller 2000; Wett et al. 2010); microwave (Kuglarz et al. 2014; Yi et al. 2014); ultrasound energy (Şahinkaya and Sevimli 2013; Zhou et al. 2015; Aylin Alagöz et al. 2018); chemical methods—ozonisation (Ak et al. 2013; Silvestre et al. 2015; Carbajo et al. 2016; Xu et al. 2018), alkalisation (Yan et al. 2013; Suschka et al. 2015; Silvestri et al. 2018a), acidification (Wang et al. 2014; Li et al. 2016; Parthiba Karthikeyan et al. 2018) and oxidation technique (Zhang et al. 2015; Lu et al. 2016; Wacławek et al. 2017; Kim et al. 2018); biological methods—enzymes (Jang et al. 2014; Kavitha et al. 2014; Lü et al. 2016; Gebreeyessus and Jenicek 2016; Ali et al. 2018) and thermal methods (heat pretreatment, freezing/thawing) (Montusiewicz et al. 2010; Carrère et al. 2010; Nowicka and Machnicka 2015; Pilli et al. 2015; Liu et al. 2018); and hybrid methods (a combination of at least two disintegration techniques) (Zhang et al. 2012; Uma Rani et al. 2014; Grübel and Suschka 2015; Suschka and Grübel 2016; Grübel et al. 2018).

Depending on the disintegration method used, there can be indicated the advantages and disadvantages of each of them. All methods cause effective destruction/disintegration which can be expressed by the so-called degree of disintegration (calculated based on the release of organic matter − soluble chemical oxygen demand (SCOD)), which increases, for example, for ultrasonic disintegration to 57.9% (Kidak et al. 2009), and for hydrodynamic to 28–35% (Grübel and Suschka 2015). Among the disadvantages of listed methods, the following could be mentioned: the demand for energy consumption and chemical reagents request, time and conditions of the processes of effective disintegration. The mentioned methods were often discussed in the review articles (Pérez-Elvira et al. 2006; Carrère et al. 2010; Tyagi and Lo 2011).

Another method for sludge pretreatment in the process of freezing presented in this article is an application of dry ice. Dry ice transforms the flocs structure into larger agglomerates and reduces bound water (Jean et al. 2001).

Dry ice or solid carbon dioxide is a completely natural product. It is produced in the form of granules, beads or prills by the compression gaseous carbon dioxide to liquid form, removing the heat generated by compression, and then rapid expansion. By expansion and rapid evaporation of carbon dioxide gas–remaining fluid, which was cooled to the melting point and frozen in the CO_2_ ‘snow’, beads or prills take form. The dry ice sublimes were at 194.65 K at a pressure of 1013.25 hPa (Jean et al. 2001; Jeyasekaran et al. 2006). Heat of sublimation is 573 kJ, which means that it is approximately 3.3 times more effective as a coolant than water ice (with the same volume). It is anhydrous, non-flammable and non-toxic and has no smell or taste (Ismalaj and Sackett 2015).

Destructive effects of low temperature on the waste-activated sludge (WAS) bind with a group of factors such as rate of freezing and thawing, chemical composition of the environment of microorganisms, species, time of freezing and temperature. However, mechanical destruction of the cells of microorganisms with the use of ice crystals, which rapture them from the inside or damage them from the outside, is put on the first place. Moreover, the process by dry ice freezing of WAS can cause the following: destruction of sludge flocs structure, increase of the volume of freezing water in the cytoplasm, mechanical damage to the wall and/or cytoplasmic membranes of microorganisms, osmotic shock, decrease of the stability of secondary structures of RNA and DNA, and reduction of the efficiency of transcription, translation, replication of DNA in cells, and ‘cold’ death of microorganisms (Seviour and Nielsen 2010).

In many technological processes, rapid heat energy introduction and dissipation is essential. Emitting or absorbing large quantities of heat during phase transformations has been used in cooling and air-conditioning devices for many years (Wiktor et al. 2015). Various media that are used are characterised by a large heat capacity, which is the ratio of specific heat to volume. One of the best substances for this purpose is water, where its specific heat is equal 4.18 kJ kg^−1^ K^−1^ and its volume is approx. 1000 kg m^−3^. However, even in this case, the amount of heat exchanged with a different factor is considerably lower than in the case of substance phase transformation (Zalba et al. 2003; Sharma et al. 2009). The method of cooling products in tavern basements with ice amassed during winter had been used for many centuries. This is why for the rapid cooling of WAS containing 92–94% water (Wójcik et al. 2017), it seems advantageous to use a substance that undergoes phase transformation at low temperatures. Dry ice is such a substance, as its sublimation temperature is approx. 194.65 K and sublimation heat is equal 573 kJ kg^−1^.

This paper focuses on the possibility of application of WAS disintegration by dry ice method and its efficiency. Herein, we have observed changes in several parameters, i.e. SCOD, proteins, RNA, PO_4_^3−^, N–NH_4_^+^ and carbohydrate concentration in the WAS liquid, and CST parameter, before and after the disintegration. Moreover, FTIR was used for detailed evaluation of change in organic content after this pretreatment.

## Materials and methods

### Waste-activated sludge samples

WAS samples were collected in 10-L containers and transported from wastewater treatment plant (WWTP), located in the Silesian voivode ship in Poland, to the laboratory of University of Bielsko-Biala. WWTP operates according to the Enhanced Biological Nutrient Removal (EBNR) processes and WAS samples were taken from outflow of secondary settling tanks. The main characteristics of WAS are as follows: pH 7.1 ± 0.2, total solids (TS) 11.81 ± 0.51 g L^−1^, volatile solids (VS) 8.16 ± 0.62 g L^−1^, soluble chemical oxygen demand (COD) 61 ± 8 mg L^−1^, total COD 9.82 ± 0.74 mg L^−1^, soluble ammonium nitrogen (N-NH_4_^+^) 2.4 ± 0.2 mg L^−1^ and soluble phosphate (P-PO_4_^3−^) 13.7 ± 0.5 mg L^−1^.

### Dry ice

Dry ice used for disintegration was in the form of prills/pellets and was obtained from the company Cryopoland sp. z o.o. Dry ice to the laboratory was transported in thermostatic containers that prevented the sublimation of dry ice.

Dry ice was produced in a pelletiser which is manufactured by taking pressurised liquid CO_2_ (horizontal storage vessel installed close to the production unit) and allowing it to expand into the natural atmosphere. This causes the liquid CO_2_ to expand into both a gas vapour and solid snow. Liquid CO_2_ is injected into chambers inside the production machines, creating pressure, and liquid CO_2_ expands; ~ 1 kg of liquid CO_2_ is flashed to make 0.45 kg of dry ice. The snow is then either compressed or extruded to form pellets. Generally, it is desirable to have dry ice pellets that are well compacted to minimise the entrapment of gaseous CO_2_ and/or air which may affect product quality. These dry ice pellets are generally in the order of 0.2 to 0.3 cm in diameter and 0.25 to 1 cm in length.

### Disintegration of WAS by freezing

Disintegration by freezing of a 1-L WAS sample was done by dry ice. The used volume ratios of the WAS to dry ice and time of disintegration (sublimation) are presented in Table 1.

**Table 1:** The used volume ratios of the WAS to dry ice and time of disintegration (sublimation)

Volume ratio of WAS to dry ice	Time of disintegration (sublimation) [hours]
1:0.25	1.5
1:0.50	2.0
1:0.75	3.0
1:1	4.5

### Methodology of phase transformation processes

In heat of phase transformations, dry ice sublimation, water solidification and also the amount of heat transferred by other substances and heat transferred from the sludge (dry sludge—without water) should be taken into account when preparing the energy balance for the process of freezing sludge. The heat absorbed from the environment during substance mixing is relatively low (according to calculations, approx. 5 kJ) and can be omitted in the overall energy balance. Exact energy balancing is extremely difficult, due to the mixing of sludge and dry ice being impeded by the rapid water solidification. Zones differing in temperature are formed, depending on the chemical composition of the specific area. Exact modelling of phase transformation processes can be found in scientific writing (Amano and Sunden 2010; Gugulothu et al. 2019). However, using the energy balance, it is possible to establish, in good approximation, the amount of dry ice needed to freeze sludge. When dry ice sublimates, it takes energy away from the sludge, causing water to freeze and bringing about the subsequent cooling of the sludge. By simplifying this process, it can be assumed that in the end stage, the temperature would be uniform in the entire volume. This assumption allows to formulate the energy balance equation, where one side contains factors attributed to dry ice (the amount of energy absorbed by dry ice when sublimating and later heating to end temperature) and the other side contains factors attributed to the sludge. The amount of heat absorbed by dry ice can be calculated using the following equation:

1 $$ {Q}_{{\mathrm{CO}}_2}={m}_{{\mathrm{CO}}_2}\cdot {c}_s+{m}_{{\mathrm{CO}}_2}\cdot \left({t}_k-{t}_s\right)\cdot {c}_{w_{{\mathrm{CO}}_2}} $$

where ${Q}_{{\mathrm{CO}}_2}$ is the heat absorbed by CO_2_; ${m}_{{\mathrm{CO}}_2}$ is the CO_2_ weight; *c*_*s*_ is the sublimation heat; *t*_*k*_ is the end temperature of mixture; *t*_*s*_ is the sublimation temperature; ${c}_{w_{{\mathrm{CO}}_2}}$ is the specific heat of CO_2_ (gas).

The amount of heat conveyed by WAS can be calculated using the following equation:

*Q*_*o*_ = *Q*_*s*_ + *Q*_*w*_ (2)

where *Q*_*o*_ is the heat conveyed by sludge; *Q*_*s*_ is the heat conveyed by solid sludge components; *Q*_*w*_ is the heat conveyed by water found in sludge.

The heat conveyed by solid sludge components which do not undergo phase transformations can be calculated using the following equation:

3 $$ {Q}_s={c}_{ws}\cdot {m}_s\cdot \left({t}_k-{t}_p\right) $$

where *c*_*ws*_ is the specific heat of solid sludge components; *m*_*s*_ is the weight of solid sludge components; *t*_*p*_ is the start sludge temperature.

The amount of heat emitted by water can be calculated based on the weight ratio of the mixed dry ice and WAS. The reason for this is that during the first stage the energy that is absorbed by the dry ice causes the cooling of the sludge. After reaching 273.15 K, the temperature stops decreasing, because the heat absorbed by the dry ice causes the phase transformation of water into ice. Only after all the water freezes does the temperature continue to decrease. Based on this, three ranges may be determined where the heat emitted by the water was calculated:

for *t*_*k*_ > 273.15 K

4 $$ {Q}_w={c}_w\cdot {m}_w\cdot \left({t}_p-{t}_k\right) $$

where *Q*_*w*_ is the heat emitted by water; *m*_*w*_ is the water weight; *c*_*w*_ is the specific heat of water;

for *t*_*k*_ = 273.15 K

5 $$ {Q}_w={c}_w\cdot {m}_w\cdot \left({t}_p-{t}_k\right)+{Q}_k $$

In this equation, it is only possible to calculate the amount of heat emitted during the solidification of water *Q*_*k*_ based on the system’s energy balance when the components’ volume fraction is known.

For *t*_*k*_ < 273.15 K

6 $$ {Q}_w={c}_w\cdot {m}_w\cdot \left({t}_p\right)+{m}_w\cdot {c}_k+{m}_w\cdot {c}_{wl}\cdot \left(-{t}_k\right) $$

where *c*_*wl*_ is the specific heat of ice; *c*_*k*_ is the heat of water solidification.

In this experiment, it is essential for the entire sludge to freeze; therefore, the minimal amount of dry ice needed for this purpose can be calculated as:

7 $$ {m}_{C{O}_2}=\frac{c_w\cdot {m}_w\cdot \left({t}_p\right)+{m}_w\cdot {c}_k+{c}_{ws}\cdot {m}_s\cdot \left({t}_p\right)}{c_s+{c}_{w_{C{O}_2}}\cdot \left(-{t}_s\right)} $$

From the equation above, it is also possible to determine the average end temperature, when the mixture composition is known. However, as it was previously mentioned, the above calculations are an approximation due to the phenomenon’s complexity; the errors are minimal. This will be shown in further calculations.

The surface of the reactors was registered using a FLIR E50 thermovision camera.

### Physicochemical analysis

In the WAS samples, before and after the process of disintegration by dry ice, the following were determined indicative of the release of organic and inorganic matter: soluble chemical oxygen demand (SCOD) value, concentration of proteins, carbohydrates, RNA acid, ammonia nitrogen, phosphates and turbidity value in the liquid phase.

SCOD, ammonia nitrogen, phosphates and turbidity value were determined for samples before and after disintegration by dry ice according to the Standard Methods for Examination of Water and Wastewater (22nd ed.) (Rice and Bridgewater 2012). The protein concentration was determined by the Lowry methods (Gerhardt et al. 1994). The carbohydrate concentration was determined by the Anthron methods (Kreith and Tchobanoglous 2002). The concentration of cellular ribonucleic acid (RNA) in the WAS samples before and after disintegration was determined by the method presented in the work of Liwarska-Bizukojc and Ledakowicz (2001). According to this methodology, a sample of WAS (5 mL) was diluted and washed three times with 3 mL cold 0.7 M HClO_4_ to destroy the cell walls of bacteria. Then, WAS was mixed with 3 mL 0.3 M KOH solution and held for 60 min at 37 °C (every 5 min, samples were mixed to hydrolyse RNA). The post-hydrolysis supernatant was collected and the precipitate was washed twice with 3 mL cold 0.5 M HClO_4_. Finally, all extracts were made up to 15 mL with 0.5 M HClO_4_ and centrifuged to remove any remaining solid particles. The obtained supernatant was subjected to the absorbance measurement (absorbance of released purines and pyrimidines) by spectrophotometer HACH DR5000 (wavelength 260 nm). The concentration of RNA was calculated according to the formula:

8 $$ {C}_{\mathrm{RNA}}=\frac{A_{260}\cdotp {M}_w}{\varepsilon l}R $$

where *M*_*W*_ = 340 g mol^−1^ is the average nucleotide molar weight; *R* is dilution; *ε* = 10,800 mol cm^−1^ is the molar absorption coefficient; *l* is the length of measuring cell (cm).

The results were taken from analyses performed at least 5 times; arithmetic average was calculated. The standard deviation was determined according to the estimator of the highest credibility in Statistica 6.0.

### The degree of disintegration

In order to obtain a quantitative measure of the effects of disintegration, the coefficient called the degree of disintegration (DD) was used for calculation according to methodology given by Müller (Müller 2000) and used earlier by our research group (Grübel and Machnicka 2013).

### Infrared waves

To confirm the release of various chemical compounds from the cells of microorganisms during the destruction of WAS by dry ice, the infrared wave analysis (IR) was applied.

A spectrometer Nicolet Magna IR 860 (Thermo Electron Corporation, USA) was used. The analysis of IR spectroscopy was carried out on suspension from evaporated liquid phase WAS samples before and after disintegration. The samples were mixed with KBr (1 mg:200 mg) and compressed in 500 MPa into pellets at 13-mm disc size; the background was a reference of pure KBr. The spectra were registered at a 4-cm^−1^ resolution in the range from 4000 to 400 cm^−1^ with 128 scans per spectrum. Data collection and post-processing were performed using OMNIC software (v. 8.0, Thermo Electron Corp.) which allows to compare obtained spectra in the scope of absorbance proportionally to the amount of the drawn sample, which allowed for quantitative interpretation of the results.

### Capillary suction time

The measurement of the capillary suction time (CST) was carried out according to Baskerville’s and Galle’s methodology based on the measurements of transition of frontal boundary layer of the filtrate as a result of the effect of suction forces in the used paper (Whatman 17).

## Results and discussion

Our investigations on the disintegration of WAS using dry ice concerned two aspects. The first was the dry ice and WAS energy balance analysis, taking into account phase transformations. The aim of this was to determine the mixture’s end temperature (with various WAS-dry ice ratio).

### The evaluation of heat phase transformations

The physical properties and thermodynamic parameters used in the calculations are presented in Table 2.

**Table 2:** The physical properties and thermodynamic parameters used in the calculations

Substance	Specific heat (J kg^−1^ K)	Solidification heat (J kg^−1^)	Sublimation heat (J kg^−1^)	Average density (kg m^−3^)
Water	4181	334,000	–	1000
Ice	2095	–	–	917
CO_2_	850 (gas)	–	573,000 (dry ice)	1300 (dry ice)
Solid sludge components (average)	1 950	–	–	1019

Theoretical amount of dry ice needed to freeze the sludge was calculated according to Eq. 7 and was 0.605 kg CO_2_.

For the mixture 1:0.25 (ratio of WAS to dry ice), the calculation showed that the amount of absorbed energy during the sublimation of dry ice and its subsequent heating to 273.15 K was approx. Q = 207 kJ, whereas to freeze the sludge, at least 387 kJ of heat emission was required. Therefore, the temperature of 273.15 K for this mixture composition was achieved, because part of the water froze and some remained in liquid state.

**Fig. 1:**
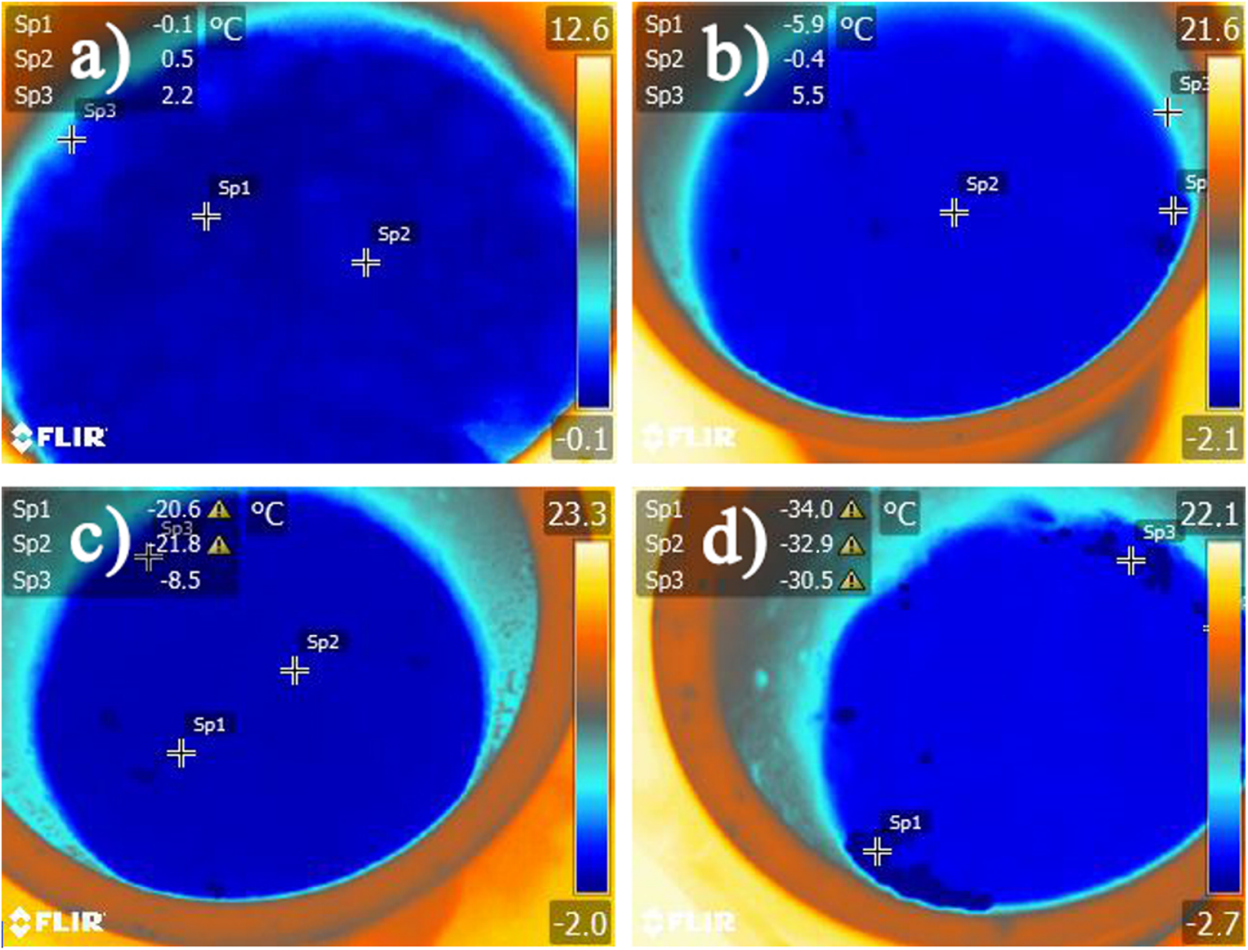
Surface temperature distribution of used mixtures. **a** 1:0.25. **b** 1:0.5. **c** 1:0.75. **d** 1:1

Calculations for the 1:0.5 ratio mixture showed that the solid carbon dioxide absorbs approx. 410 kJ of energy; therefore, the temperature of the mixture should be around 263.15 K. The temperature of 267.15 K (Fig. 1b) was determined after taking into account the average amount of heat absorbed during wall condensation caused by air humidity (when air humidity equals 0.1 kg H_2_O kg_p.s_^−1^) and that part of the CO_2_ is released and leaves the system, only partially absorbing heat form the sludge (80% of theoretical amount of absorbed heat). The surface of the mixtures ratio (WAS-dry ice) was registered using a FLIR E50 thermovision camera and is shown in Fig. 1.

Calculations for the 1:0.75 ratio mixture showed that the CO_2_ absorbs approx. 558 kJ of energy. The mixture should therefore reach the temperature equal to the temperature of dry ice sublimation. The amount of absorbed heat during the sublimation transformation of such an amount of dry ice causes the freezing of sludge and then its further cooling to approximately 194.65 K. Theoretical calculations assume temperature levelling in the entire mixture volume. This is theoretically possible only after accurate isolation of the mixture and complete sublimation of the dry ice. In practice, it is not possible to reach this state, due to the forming of an ice coat around the fragments of dry ice, which hinders the sublimation process. Despite this, in Fig. 1c, it is possible to observe temperature points that go beyond the range of the thermovision camera.

Even considerably higher temperature points were also found. These are found on the edges of the container and on larger ice fragments. It can be assumed that if the system was left in isolation longer (not frozen at once), the temperature would be much more uniform in the entire mixture volume.

Calculations for the 1:1 ratio mixture showed that the CO_2_ absorbs approximately 745 kJ of energy. As was in the previous case, the amount of absorbed energy is enough to reach the sublimation temperature, which is ca. 194.65 K. Similarly as in the previous case, it is impossible to reach this temperature in the entire volume in a short space on time due to the heterogeneity within the mixture’s volume (solid and liquid state of WAS). Large areas going beyond the camera’s range can be observed on the thermovision image (Fig. 1d). The application of a mechanical stirrer capable of crumbling the formed ice fragments would cause quicker uniform sludge cooling.

We used variable volumetric values of dry ice to WAS. This methodology contributed to the different time of disintegration of the WAS. A small proportion of dry ice (1:0.25) did not cause complete freezing of the entire sample volume and the thawing was fastest (this was also confirmed by observation using a thermal imaging camera and calculations of heat phase transformations). A dose of 1:0.75 and higher resulted in complete freezing of the samples and effective disintegration lasting 3–4.5 h.

### Changes in the chemical composition of supernatant

During the next stage, we tried to evaluate the effect of the freezing phenomenon on the release of chemical compounds.

Disintegration of WAS by dry ice caused the release of organic matter (expressed as SCOD) to a supernatant of the sludge. Changes in SCOD were associated with the disruption of the WAS flocs and cells of microorganisms, which led to the release of organic compounds to the sludge liquid.

**Fig. 2:**
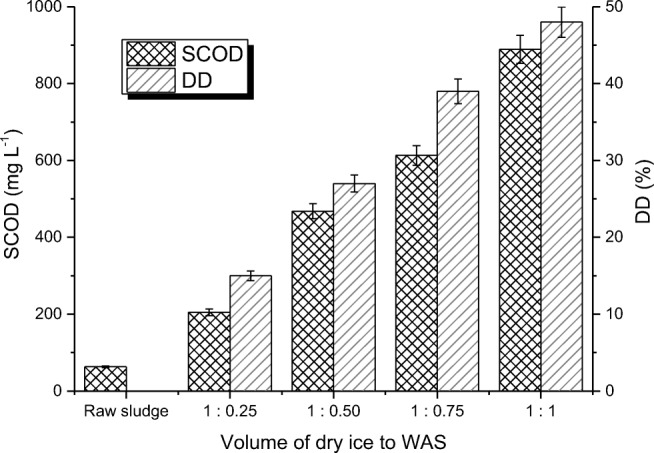
Changes soluble chemical oxygen demand (SCOD) value and degree of disintegration (DD) before and after the disintegration of sludge by dry ice

During the WAS disintegration by dry ice, the intensification of increase of SCOD was indicated, depending on the applied volume ratio of sludge to dry ice. Disintegration by dry ice for the volume ratio of the sludge to dry ice 1:0.25 contributed to the increase of SCOD from the initial value of 63 mg O_2_ L^−1^ (raw sludge) to 205 mg O_2_ L^−1^ (Fig. 2) and by increasing the dose of dry ice in relation to the sludge in a ratio of 1:1, SCOD achieved the value of 889 mg O_2_ L^−1^ (Fig. 2).

Chen et al. (2014) studied the effectiveness of freezing of sludges in order to increase degradation of organic substances to the benefit of growth in biogas production and the use of microbiological substrate in the fuel cell (MFC) to produce electric energy. The obtained results demonstrated that a long time of sludge freezing (more than 48 h) resulted in release of substrate, which is susceptible to biochemical degradation process and provided the possibility of its processing into electric energy. The obtained about 40.9% higher removal efficiency of total SCOD and increase of maximum power output about 1.3 W m^−3^ in MFC.

The intracellular substances were released to the liquid phase as a result of crystallisation of flocs and microorganisms cells. Release of organic matter in the process of freezing was examined by Hu et al. (2004). They obtained more than 15% increase of the SCOD value.

Wang et al. (1999) compared the effects of thermal sonolysis with the effects of freezing in − 10 °C temperature and sonification. They found that the increase of the dissolved SCOD was higher for 40 min of sonification and freezing to − 10 °C temperature, while only sonification appeared to be ineffective. Therefore, one of the assumptions could be that freezing of sludge has a positive impact on the release of organic matter to a solution (however, it should be mentioned that the authors in article focused mainly on the combined pretreatment).

As expected, with the increase of the dose of the solid carbon dioxide CO_2_ in relation to the WAS, the disintegration degree of samples (DD) increased.

As a result of destructive effects of dry ice to the WAS structure for the volume ratio of WAS to dry ice 1:0.25, the DD increased to 15% (Fig. 2). A further increase of the volume of dry ice doses in relation to the WAS (the volume ratio of WAS to dry ice 1:0.5; 1:0.75; 1:1) resulted in a further increase of the DD to 48% (Fig. 2).

A significant release of proteins was analogous to the increase in the value of SCOD. The increase in proteins concentration was related to the destructive effect of low temperatures on the cells of microorganisms and introducing enzymes and structural proteins to the solution (Örmeci and Aarne Vesilind 2001). The proteins concentration in the liquid phase of WAS increased from 56 mg L^−1^ (raw sludge) to 99 mg L^−1^ for the volume ratio of WAS to dry ice 1:0.25 (Fig. 2). A further increase of WAS to dry ice ratios caused progressive increase of proteins concentration in the solution to 291 mg L^−1^ (Fig. 2).

**Fig. 3:**
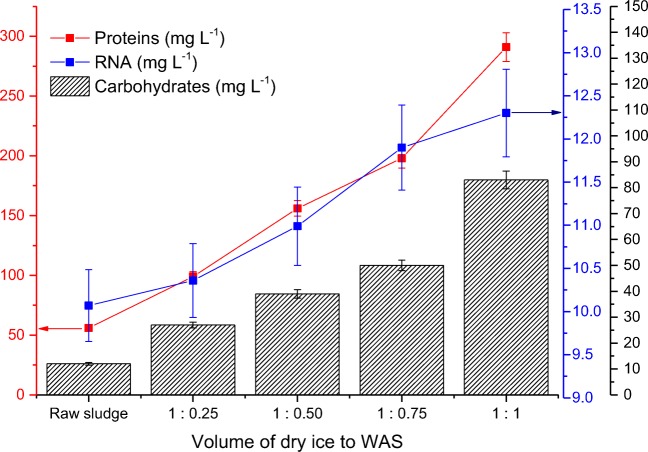
Changes of proteins, carbohydrate and RNA concentration before and after disintegration of WAS by dry ice

Thus, disruption of WAS flocs and cells of microorganisms by disintegration by dry ice resulted in release of organic matter to the liquid sludge phase, expressed in the increase of the SCOD value, concentration of proteins and carbohydrates (Figs. 2 and 3).

The effectiveness of the process of freezing of WAS has been also expressed by change in the concentration of carbohydrates in the supernatant of the WAS. Disintegration of the WAS by dry ice led to an increase of carbohydrate concentration in the liquid phase, from the initial value of 12 mg L^−1^ for raw WAS to the value of 83 mg L^−1^ for the volume ratio of WAS to dry ice of 1:1 (Fig. 3).

The impact of freezing on the release of organic and inorganic substances (for example, proteins, carbohydrates, phosphates) from the microorganisms cells was also shown by other researchers (Zaia et al. 2000; Örmeci and Aarne Vesilind 2001; Montusiewicz et al. 2010; Gao 2011; Hu et al. 2011).

Determining the origin of chemical compounds components of biomass in a liquid sewage sludge phase is often very difficult. Therefore, another compound, which confirms the destruction of cells of microorganisms is RNA (ribonucleic acid) in the sludge liquid. In the last decade, a number of researchers used Schneider’s method with the use of hot perchlorid acid to determine the RNA or DNA concentration in a medium. Another method used in the studies is a technique which uses cold HClO_4_ and spectrophotometer (Liwarska-Bizukojc and Ledakowicz 2001).

Based on the conducted analysis on the effective action of dry ice on the microorganisms of the WAS, it has also been found that the increase of the dose of dry ice in relation to the sludge was followed by an increase of the concentration of RNA acid in the liquid of sludge samples. For the control sample (raw sludge), the concentration of RNA amounted to 10.07 mg L^−1^, while for the volume ratio of sludge to dry ice 1:1, the concentration of RNA increased by 2.23 mg L^−1^ (Fig. 3).

As a result of destruction of cells structure of microorganisms by dry ice, the release of enzymes contained in the protoplast of microorganisms took place, whose effect of hydrolytic action was decomposition of nitrogen and phosphorous organic compounds and thus, there was also an increase of concentration of ammonium nitrogen and phosphates in liquid phase.

**Fig. 4:**
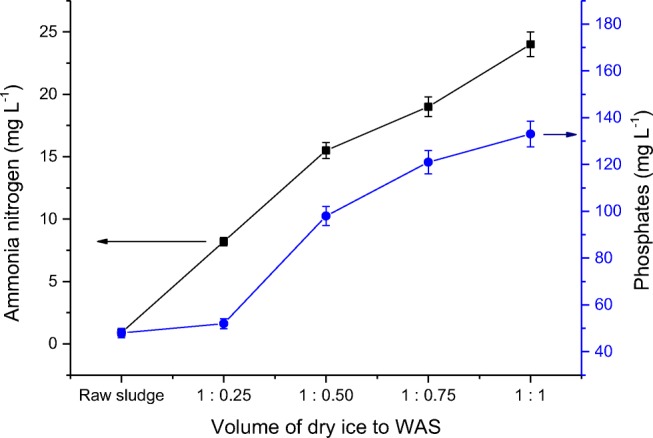
Changes of ammonium nitrogen and phosphates concentration before and after disintegration of WAS by dry ice

The dry ice action on the WAS resulted in gradual increase of ammonium nitrogen concentration in liquid and at the volume rate of WAS to dry ice of 1:1; the N–NH_4_^+^ concentration amounted to 24 mg L^−1^ (Fig. 4).

The process of freezing of WAS by dry ice contributed to the increase of concentration of phosphates with an initial value of 4.8 mg PO_4_^3−^ L^−1^ (raw sludge) to 133 mg PO_4_^3−^ L^−1^ (the volume ratio of WAS to dry ice 1:1) (Fig. 4).

In 2011, Gao (2011) conducted laboratory tests of pretreatment of WAS, comparing freezing (− 18 °C) of WAS with chemical methods (acidification: pH 5.5, alkalisation: pH 11.0) and thermal (60 °C). He showed that freezing of WAS is an effective disintegration method, which resulted in an 8-fold increase of SCOD and ammonia nitrogen and 2.5-fold increase of phosphates.

Montusiewicz et al. (2010) evaluated the effect of disintegration of sludge’s via (using variable temperature) freezing on the properties of mixed sludge (real and waste) from municipal wastewater treatment plant. They examine the effects of the process of freezing on the quality of supernatant. The effect of freezing caused a 10% increase of concentration of nitrogen and phosphate compounds, which corresponds with the results obtained to some extent.

The obtained results confirm effective destruction of sludge flocs and ‘cold’ death of microorganisms caused by e.g. speed and time of freezing and thawing, chemical composition of sludge, bacterial species and temperature. For the mechanical destruction of the cells, in this destruction method, ice crystals are responsible, which damage them from the outside or inside (Örmeci and Aarne Vesilind 2001). The effect of this is the leakage of cellular components (organic and inorganic) over the shields into the liquid phase of sludge. In addition, the particular susceptibility to cold shock is mainly demonstrated by Gram-negative bacteria (they are present in large quantities in WAS) and those that are at the logarithmic growth stage (Vollmer 2004).

**Fig. 5:**
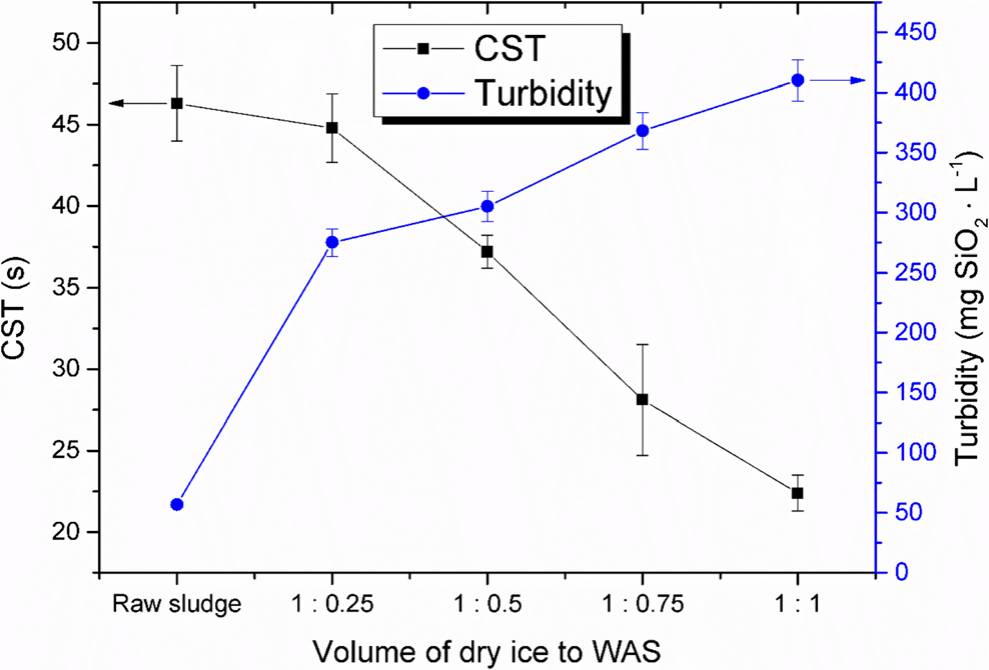
Changes in the turbidity and capillary suction time (in the liquid phase) before and after disintegration of WAS by dry ice

Disintegration of WAS by dry ice led to an increase of turbidity of supernatant, which was caused by adding the suspension load and organic and inorganic colloids to the solution. The turbidity increased from the initial value of 57 mg SiO_2_ L^−1^ for a raw sludge to the value of 410 mg SiO_2_ L^−1^ for the volume ratio of sludge to dry ice 1:1 (Fig. 5). Its changes were dependent on the volume ratio of sludge to dry ice, which was applied (Fig. 5).

Introduction of thermal WAS pretreatment in previous years aimed at improving the degree of mineralisation of sludge as well as its dewatering (Örmeci 2004; Diak et al. 2011). The dewatering of sewage sludges with the use of freezing takes place through separation of solid and liquid fractions during the formation of ice crystals. It was also found that this mechanism favours transformation of sediment flocs in agglomerates in a more compact and dense form (Hung et al. 1996; Jean et al. 2001).

The capillary suction time (CST) can be used as a good index for the ability to dewater of sludge. The test measures the time for free water to pass through a certain distance using suction paper as the medium (Chen et al. 1996). In this study, the dry ice action on the WAS resulted in decrease of CST from 46.2 to 22.8 s at the volume rate of WAS to dry ice of 1:1.

Wang et al. (2001) obtained over 82% increase of sludge dewaterability after sludge freezing compared with the untreated sludge. Additionally, they noted seven times better sludge dewaterability when slow-frozen process was applied in comparison with fast-frozen process.

**Fig. 6:**
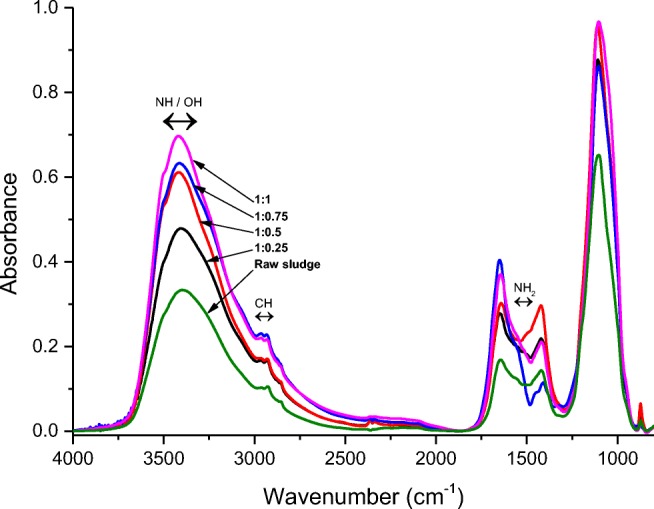
Individual wavelengths characteristic for oscillators of chemical groups for freezing WAS disintegration

The confirmation of physicochemical changes in supernatant was provided by an infrared spectroscopy research. The research results obtained confirm an increase of released and dissolved intracellular substances (Fig. 6).

IR is a technique used to obtain oscillatory and rotatory spectra. IR spectroscopy enables to establish which functional groups are present in an analysed sample. Infrared spectroscopy also enables particle structure analysis and their interaction with the environment. Two different chemical compounds will not have identical spectra in entire IR range and this ability allows for unambiguous identification of compounds.

Changes in the values of chemical parameters of liquid involved in the WAS depended on the volume ratio of WAS to dry ice which was applied. Following the increase in the volume of dry ice dose to WAS, greater changes in designated concentrations appeared. Considering influence of the process of freezing, it was found out that changes in chemical properties appeared in the samples due to the lysis of cells and release of enzymes. The use of thermal phenomena caused physicochemical reactions that have influenced the nature of distribution of substances present in the supernatant. Consequently, this led to the release of organic and inorganic matter and inter- and extracellular enzymes appearing in connections with colloid substances.

As was mentioned earlier, an increase in absorbance for individual wavelengths is very unique and characteristic for oscillators of chemical groups. The obtained results confirmed the differences in the liquid phase composition after application of WAS freezing (increased with the volume ratio of WAS to dry ice).

Analysing the changes in absorbance at a specific wavelength confirmed increase of the concentration of oscillators of tensile vibration for O–H and N–H compounds (at wavelengths around 3400 cm^−1^), S–H and C–H (at wavelengths around 2900 cm^−1^, 3000 cm^−1^ and 1400 cm^−1^), C=O (at wavelengths around 1650 cm^−1^) and C–O, P–O and S–O (at wavelengths around 1100 cm^−1^) (Fig. 6) (Socrates 2007). These compounds are buildings cells of microorganisms and changes of them concentrations in liquid phase (suspensions) confirm the effective disintegration of applied freezing process.

The mentioned changes in absorbance at the specific wavelength are indicative of the release of i.e. amines, amino acids, proteins, phosphates, etc. Revealing these chemical groups in the supernatant attests to a destructive influence of freezing on WAS microorganisms and effective cellular lysis.

Dry ice in increased concentration caused further transformation of sludge what was revealed by the change in the intensity ratio of bands 1650 and 1420 cm^−1^. This can be related to the amine group transformation after the dry ice treatment (Silvestri et al. 2018b).

Moreover, there is a proportionally lesser absorbance increment for the 1:0.25 WAS to dry ice volume ratio than the 1:1 ratio. A smaller value in absorbance increment for the 1:0.25 ratio may have occurred due to the onset of homogenisation of the disintegrated medium, whereas the disintegration only increases for the 1:1 ratio.

The impact of the process of freezing by dry ice of sewage sludge on the increase of concentrations of organic and inorganic matter, determined by spectroscopic analysis, was confirmed using the IR analysis, which corresponds with the results obtained by Hu et al. (2011).

## Conclusions

Calculations showed that the dry ice-WAS mixture, ratio 1:0.25, achieved the temperature 273.15 K, whereas the mixture of solidified carbon dioxide and WAS, ratio 1:0.5, reached 267.15 K. In mixtures, with the ratios of 1:0.75 and 1:1, the CO_2_ absorbed 558 kJ and 745 kJ of heat, respectively. This resulted in achieving mixtures that reached the temperature of dry ice sublimation. Moreover, as a result of disintegration of WAS by dry ice (in all ratios), a destruction of cellular structures of microorganisms appeared, and thus, there was an increase in SCOD, proteins, RNA, PO_4_^3−^, N–NH_4_^+^ and carbohydrate concentration in the sludge liquid. Furthermore, disintegration of WAS by dry ice caused an increase in the turbidity of supernatant and at the same time CST decreased which can be used as a good index for the ability to dewater WAS. FTIR analysis has confirmed the hypothesis of an efficient disintegration of WAS. That could be concluded from the changes in absorbance at the specific wavelengths, which corresponded to the presence of among others, amines, amino acids, proteins and phosphates. The separation of these substances in the supernatant confirms the destructive action on microorganisms (localised in WAS) and effective lysis of microorganisms cells.

## Electronic supplementary material

ESM 1 (DOCX 233 kb)
