# Supplementary material for: Waste-activated sludge disruption by dry ice: bench scale study and evaluation of heat phase transformations
Source: Environ Sci Pollut Res Int. 2019 Jul 9;26(26):26488–99. doi: 10.1007/s11356-019-05889-2 (PMC6733821; doi:10.1007/s11356-019-05889-2)
Supplement: Supplementary file 1 — (DOCX 233 kb) [file 11356_2019_5889_MOESM1_ESM.docx]

Supplementary data:

***Microscopy analysis***

Biological changes of the sludge were determined on the basis of variations in the structure of the WAS flocs using an electron scanning microscope with a rengenographic adapter of energy disperse (EDX – *Energy Dispersive analysis of X – rays attachment).* In this microscope, a strongly focused electron beam passes through following layers of the object. Emitted electrons are focused by the collector, which controls the electron beam running synchronously in the electron tube. This beam catches the luminescent screen and produces a magnified image of the object, and the EDX adapter enables the study of chemical structure of an object of interest.

Confirmation of physicochemical results obtained were microscopy analysis with the use of the electron scanning microscope, which were carried out.

In order to maximise the release of organic and inorganic substance from the cells of microorganisms, it is aimed to obtain the particles of possibly the smallest sizes in disintegration processes.

Under laboratory conditions, due to performed thermal disintegration of microorganisms of WAS, a variable particle size has been obtained, however, not greater than 0.4 mm. The structure of the obtained mixture and sizes of particles achieved, for the volume ratio of WAS to dry ice 1:1, is presented on Fig. A.


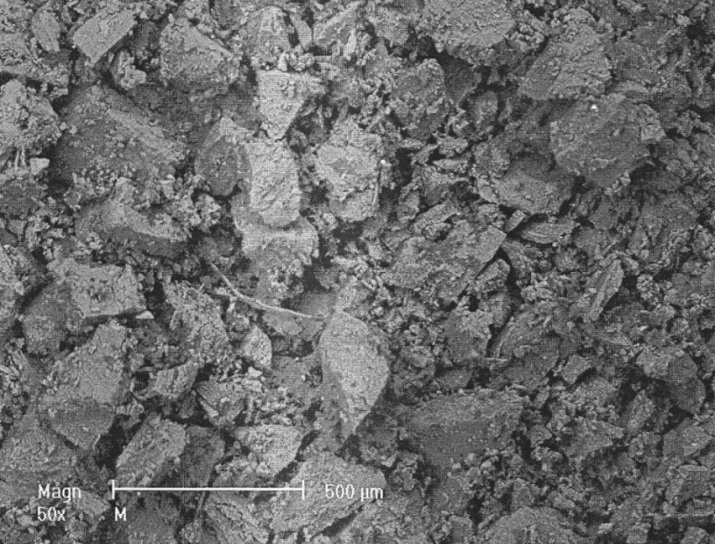


**Fig. A.** Disintegrated WAS (the volume ratio of WAS to dry ice - 1:1) – a picture from the electron scanning microscope
